# Collective cell migration of fibroblasts is affected by horizontal vibration of the cell culture dish

**DOI:** 10.1002/elsc.202000013

**Published:** 2020-07-19

**Authors:** Umi Enomoto, Chikahiro Imashiro, Kenjiro Takemura

**Affiliations:** ^1^ School of Science for Open and Environmental Systems Graduate School of Science and Technology Keio University Yokohama Kanagawa Japan; ^2^ Department of Mechanical Engineering Keio University Yokohama Kanagawa Japan; ^3^ Institute of Advanced Biomedical Engineering and Science Tokyo Women's Medical University Tokyo Japan

**Keywords:** cell migration, fibroblast, mechanotransduction, vibrational stimulation, wound healing

## Abstract

Regulating the collective migration of cells is an important issue in bioengineering. Enhancing or suppressing cell migration and controlling the migration direction is useful for various physiological phenomena such as wound healing. Several methods of migration regulation based on different mechanical stimuli have been reported. While vibrational stimuli, such as sound waves, show promise for regulating migration, the effect of the vibration direction on collective cell migration has not been studied in depth. Therefore, we fabricated a vibrating system that can apply horizontal vibration to a cell culture dish. Here, we evaluated the effect of the vibration direction on the collective migration of fibroblasts in a wound model comprising two culture areas separated by a gap. Results showed that the vibration direction affects the cell migration distance: vibration orthogonal to the gap enhances the collective cell migration distance while vibration parallel to the gap suppresses it. Results also showed that conditions leading to enhanced migration distance were also associated with elevated glucose consumption. Furthermore, under conditions promoting cell migration, the cell nuclei become elongated and oriented orthogonal to the gap. In contrast, under conditions that reduce the migration distance, cell nuclei were oriented to the direction parallel to the gap.

## 1 INTRODUCTION

Collective cell migration defined as the movement of multiple cells that retain cell–cell contacts [1] is involved with various physiological phenomena such as wound healing, cancer infiltration, embryonic development, and embryogenesis [2, 3]. Enhancing or suppressing cell migration and controlling the migration direction may be useful for wound healing, suppressing cancer metastasis, controlling tissue formation, and other applications [4, 5]. Therefore, the regulation of collective cell migration is one of the most important issues in bioengineering and biomedical research.

Since collective cell migration is known to be affected by external stimuli, a number of methods have been developed to regulate cell migration by applying different stimuli such as chemical, electrical, and mechanical stimuli [6, 7, 8]. Mechanical stimuli have recently gained attention as they are known to affect several crucial factors related to migration, including cell adhesion and morphology [9]. The mechanism by which mechanical stimuli regulate migration is described briefly as follows: focal adhesions perceive mechanical stimulus [10] and initiate signaling to the cell nucleus via the cytoskeleton network; then, actin remodeling is initiated, giving rise to the traction forces needed for cell migration [11]. This signal transduction in response to mechanical stimuli via biochemical signals in a cell is called mechanotransduction [12].

Various methods can be used to apply mechanical stimuli to cells. For example, cells can be seeded on a silicone elastomer that is cyclically stretched and relaxed to apply a periodic stretch stimulus [13]. Fluid flow through a microfluidic device applies shear stress to cells cultured in the device [14]. Vibrational stimulation is gaining attention because it is easy to apply in the ubiquitous cell culture dish format and is highly biocompatibile [15]. The method using vibration stimulation has the advantage that the culture environment is not changed, compared with the conventional migration regulation method represented by using chemical stimulation. Vibration stimulation is widely used in vivo, for example, as ultrasonic therapy, and its biocompatibility is clear. In vitro, it has been reported that proper acoustic vibration applied from above or below a cell‐culture dish enhances migration distance [16, 17]. However, vibrational stimuli have only been applied in a direction orthogonal to the culture surface to regulate migration, and the effect of the vibration direction on the migration direction has not been investigated. Since migration involves collective directional movement, the directionality of the stimulus is considered important [2]. For instance, when exposed to a stretch stimulus, cells tend to arrange and migrate in parallel to the direction of the extension [18]. Besides this, it has been reported that horizontal and vertical vibrations have different effects on proliferation and actin organization [19]; however, the effect on cell migration has not been discussed. Since cells migrate along a surface, we hypothesize that vibrational stimuli should be applied in a direction along the culture surface to regulate migration direction. A new migration regulation method using horizontal vibration may affect the direction of cell migration, unlike the conventional method using vertical vibration.

The objective of this study is to clarify the effect of the stimulus direction on cell migration. To experimentally evaluate this relationship, we fabricated a vibrating system that can apply horizontal vibration to cells by horizontally oscillating a ⌀35 mm cell culture dish. Here, we evaluate the effect of different horizontal vibrations on the collective migration of fibroblasts in a wound model created by the solid barrier method [20]. Cell orientation and glucose consumption were also evaluated.

**PRACTICAL APPLICATION**

Regulating migration is useful for wound healing, suppressing cancer metastasis, controlling tissue formation. Previous studies reported that cell migration is affected by vertical vibration. However, the effect of horizontal vibration on cells has not been reported. In this study, we observed that when cells in a wound model were subjected to horizontal vibration stimulus, the distance of collective migration, glucose consumption, and nucleus shape and orientation were affected by the direction of the vibration stimulus. The overall results suggest the importance of the stimulus direction in regulating migration by using vibration stimulus. This study potentially represents novel application of wound healing by using a vibration stimulus.

## 2 MATERIALS AND METHODS

### 2.1 Vibrating system

**FIGURE 1:**
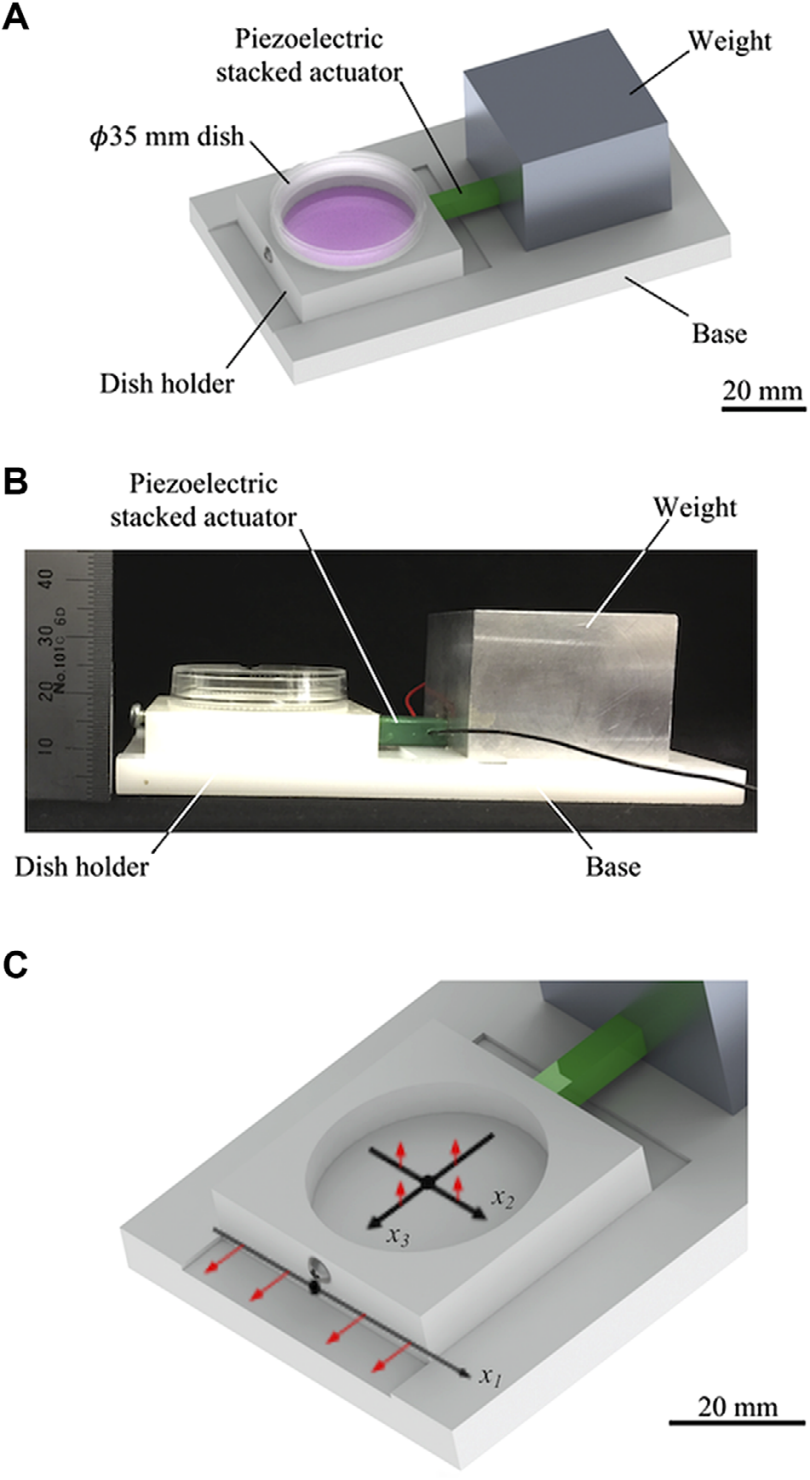
The fabricated system applies horizontal vibration to the cells: (A‐B) Overview of the system. (C) Enlarged view of the dish holder and coordinate system. The red arrows indicate the direction of the vibration measurements conducted for system characterization

The developed vibrating system is shown in Figure 1A. The system comprised a base, a dish holder, a ⌀35 mm cell culture dish (Nunc™ EasYDish™ 35 mm, Thermo Fisher Scientific, MA, USA), a piezoelectric stacked actuator (PSt 150hTc/5 × 5/18, Syouei System Co., Ltd. Tokyo, Japan), and a weight. The culture dish was placed on the dish holder and fixed with a stainless steel screw. The dish holder and base were made of polyacetal, which has a friction coefficient that is low enough to vibrate the dish efficiently. The weight was fixed to the base. The piezoelectric stacked actuator was sandwiched between the dish holder and the weight. An AC voltage was applied to cause the actuator the periodically expand and contract along the longitudinal direction, thereby vibrating the dish on the horizontal plane. A piezoelectric actuator was selected because it is available in kHz‐range actuation. If a dish is vibrated in Hz‐range, sloshing of the culture medium may apply shear stress to cells. In addition, heat generation may become a problem when using MHz‐range vibration.

### 2.2 System characterization

The system vibration characteristics were evaluated as follows: an AC voltage was generated by a function synthesizer (WF1946B, NF Corp., Kanagawa, Japan) and an amplifier (HSA4011, NF Corp., Kanagawa, Japan) and applied to the actuator. The vibration amplitude of the dish holder fitted with an empty dish was calculated from the vibration velocity measured using a laser Doppler vibrometer (LV‐1800, Ono Sokki Co. Ltd., Kanagawa, Japan). The relationship between the applied voltage and the vibration amplitude of the dish holder was estimated by measuring the vibration amplitude at the position *x_1_* = 0, as defined in Figure 1B. The frequency of the applied voltage was 11.2 kHz, and the amplitude of the applied voltage was varied from 0 to 5 V. The distribution of the vibration amplitude was then measured along the *x_1_*, *x_2_*, and *x_3_* axes in the dish holder in the directions indicated by the red arrows in Figure 1B when the frequency and voltage were 11.2 kHz and 2.5 V, respectively. Measurements were conducted at points from –10 to 10 mm at 1 mm intervals along each axis.

The temperature of the medium (1 mL) in the culture dish was measured using a thermometer (TR‐71wf, T&D Corporation, Nagano, Japan) over the course of 24 h while the system was running in a humidified 5% CO_2_ incubator (CPE‐2201, Hirasawa Works Inc., Tokyo, Japan) maintained at 37°C. The frequency of the applied voltage was 11.2 kHz, and the amplitude was 2 or 4 V.

### 2.3 Cell preparation

A mouse fibroblast cell line, L929 (RCB1451, Riken Bio Resource Center, Ibaraki, Japan), was used in all experiments. The cells were cultured in Eagle's minimal essential medium (Eagle's MEM “Nissui”, Nissui Pharmaceutical Co., Ltd., Tokyo, Japan) supplemented with 5% calf serum (Newborn Calf Serum, New Zealand Origin, Thermo Fisher Scientific, MA, USA) in a humidified 5% CO_2_ incubator at 37°C. Cell passaging was performed by applying 0.05% trypsin‐EDTA (25300, Life Technologies, CA, USA) followed by pipetting.

### 2.4 Collective cell migration experiment

**FIGURE 2:**
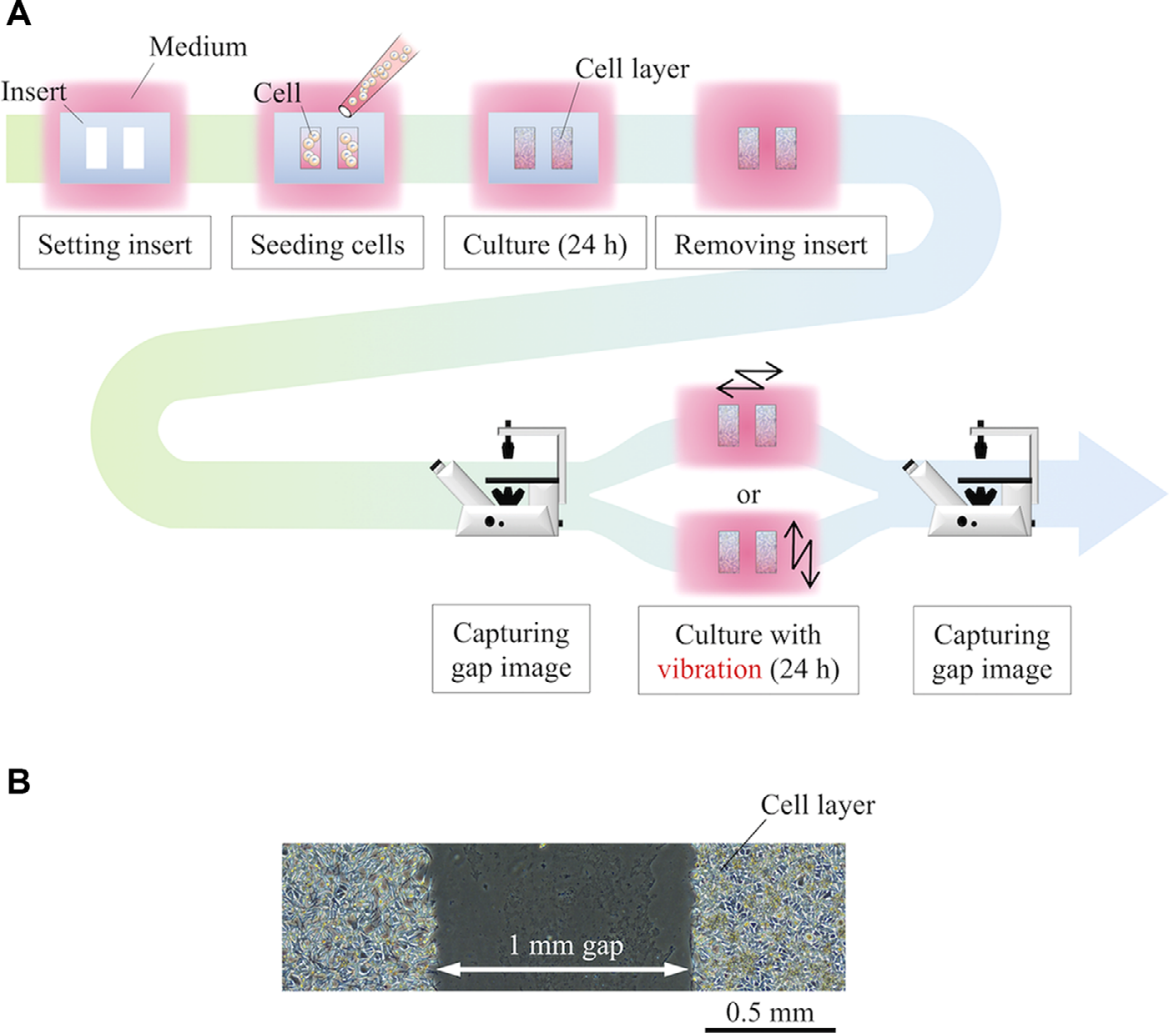
Outline of the collective cell migration experiment: (A) Experimental procedure and (B) initial gap between the two rectangular cell areas

The experimental procedure is shown in Figure 2A. First, 1.0 × 10^4^ cells in 10 µL of medium were applied in each of two rectangular areas (3 mm × 1.5 mm) in a cell culture dish. The two culture areas were separated by a 1 mm gap by using a silicone rubber insert. The cells were then cultured for 24 h in a humidified 5% CO_2_ incubator at 37°C to allow the cells to attach. After the initial incubation period, the insert and medium were removed, and a phase‐contrast image of the gap was captured using an inverted microscope (Eclipse Ti, Nikon Corp., Tokyo, Japan). The initial 1 mm gap between the two cell culture areas is shown in Figure 2B. Then, 1 mL of medium was added to the dish, and the cells were cultured for 24 h in a humidified 5% CO_2_ incubator at 37°C with vibration. The frequency of the applied voltage was 11.2 kHz and the amplitude was 0 (i.e. no vibration), 2, or 4 V. To evaluate the effect of the vibration direction on cell migration, the cells were subjected vibrational stimuli either orthogonal or parallel to the gap. Finally, the gap between the two culture areas was imaged for a second time, and the size of the gap was measured. For the measurement, the average distance between the edges along the horizontal direction in the images was manually calculated with an image processing software, Image J (National Institutes of Health, Bethesda, MD, USA). The migration distance was then calculated as the difference in the gap size before and after the stimulation.

### 2.5 Glucose consumption assay

The glucose consumption by the cells with and without vibration was monitored over a 24 h period. Since migration is an activity that consumes glucose, glucose consumption can be an indicator for the evaluation of cell migration. The amounts of glucose contained in the medium in which the cells were cultured and a control sample of medium that was not exposed to cells were measured using a glucose assay kit (GAHK‐20, Sigma‐Aldrich, MO, USA) and a microplate reader (Multiskan FC, Thermo Fisher Scientific, MA, USA). The glucose consumption was calculated by comparing the measured glucose concentrations of the culture medium taken from the culture dish and the control medium sample.

### 2.6 Cell staining and image‐based quantification

The morphologies of cell nuclei with and without vibrational stimulation were observed from fluorescent images of the cells. After removing the medium, cells were washed three times with 1 mL PBS. Next, cells were fixed with 4% paraformaldehyde (4% – Paraformaldehyde Phosphate Buffer Solution, Nacalai Tesque, Inc., Kyoto, Japan) in PBS for 10 min, washed with PBS, permeabilized with 0.1% Triton X‐100 (Triton X‐100 laboratory grade, Sigma‐Aldrich, MO, USA) in PBS for 5 min, and finally washed with PBS. After removing the PBS, the cells were stained with 0.05% Hoechst 33342 (H342, Dojindo Laboratories, Kumamoto, Japan) for 30 min and again washed with PBS. Then, fluorescence images of the cells were captured using the inverted microscope.

The captured fluorescent images were analyzed by fitting an ellipse to each cell nucleus using Image J. 300 cell nuclei near the gap edge were randomly selected to be analyzed. The angle of each cell nucleus was measured as the angle between the major axis of the fitted ellipse and a straight line parallel to the gap. The aspect ratio of each nucleus was measured as the ratio of the major axis to the minor axis of the fitted ellipse.

### 2.7 Statistical analysis

The differences between cells exposed to different conditions were evaluated by non‐parametric analysis of variance with multiple comparisons using Bonferroni's method. *p* < 0.05 was considered to indicate statistical significance.

## 3 RESULTS

### 3.1 System characterization

**FIGURE 3:**
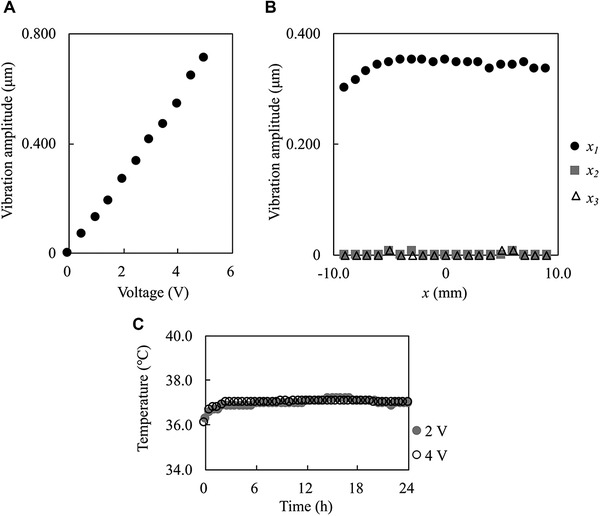
Characteristics of the fabricated system: (A) Relationship between the applied voltage and the vibration amplitude at *x_1_* = 0 under an applied frequency of 11.2 kHz and amplitude of the applied voltage was 0 to 5 V. (B) Vibration amplitude distribution of the dish holder along the *x_1_*, *x_2_*, and *x_3_* axes when the frequency and amplitude of the applied voltage were 11.2 kHz and 2.5 V, respectively. (C) Temperature of the culture medium in the dish vibrated by the fabricated system for 24 h in a humidified 5% CO_2_ incubator at 37°C when the frequency of the applied voltage was 11.2 kHz and the voltage amplitude was 2 or 4 V

The relationship between the applied voltage and the vibration amplitude of the dish holder at the position *x_1_* = 0 (cf. Figure 1B) is shown in Figure 3A. The data show that the vibration amplitude of the dish holder is proportional to the applied voltage. From this result, the acceleration of the cells when the applied voltage is 2/4 V is about 136/272 *G*_peak_.

The vibration amplitude distribution of the dish holder along the *x_1_*, *x_2_*, and *x_3_* axes (cf. Figure 1B) is shown in Figure 3B. These data show that the vibration amplitude of the dish holder along the *x_1_* axis is approximately constant in the range of about 10 mm from the center. Since the cells were seeded within about 2 mm from the center, it was concluded that the experimental setup could apply uniform oscillations to the cultured cells. The data also show that the vibration amplitude of the dish holder in the vertical direction is small enough to be disregarded. Therefore, it can be concluded that this experimental system oscillates the cells in the dish only on the horizontal plane.

The temperature of the culture medium in the dish while the system was operated for 24 h is shown in Figure 3C. With applied voltages of 2 and 4 V, the temperature of the medium was stable at 37°C and not affected by the vibration. Since the temperature suitable for cell culture is 36–38°C, it was concluded that the temperature during the vibrating experiments was suitable for cell culture [21].

### 3.2 Evaluation of collective cell migration

**FIGURE 4:**
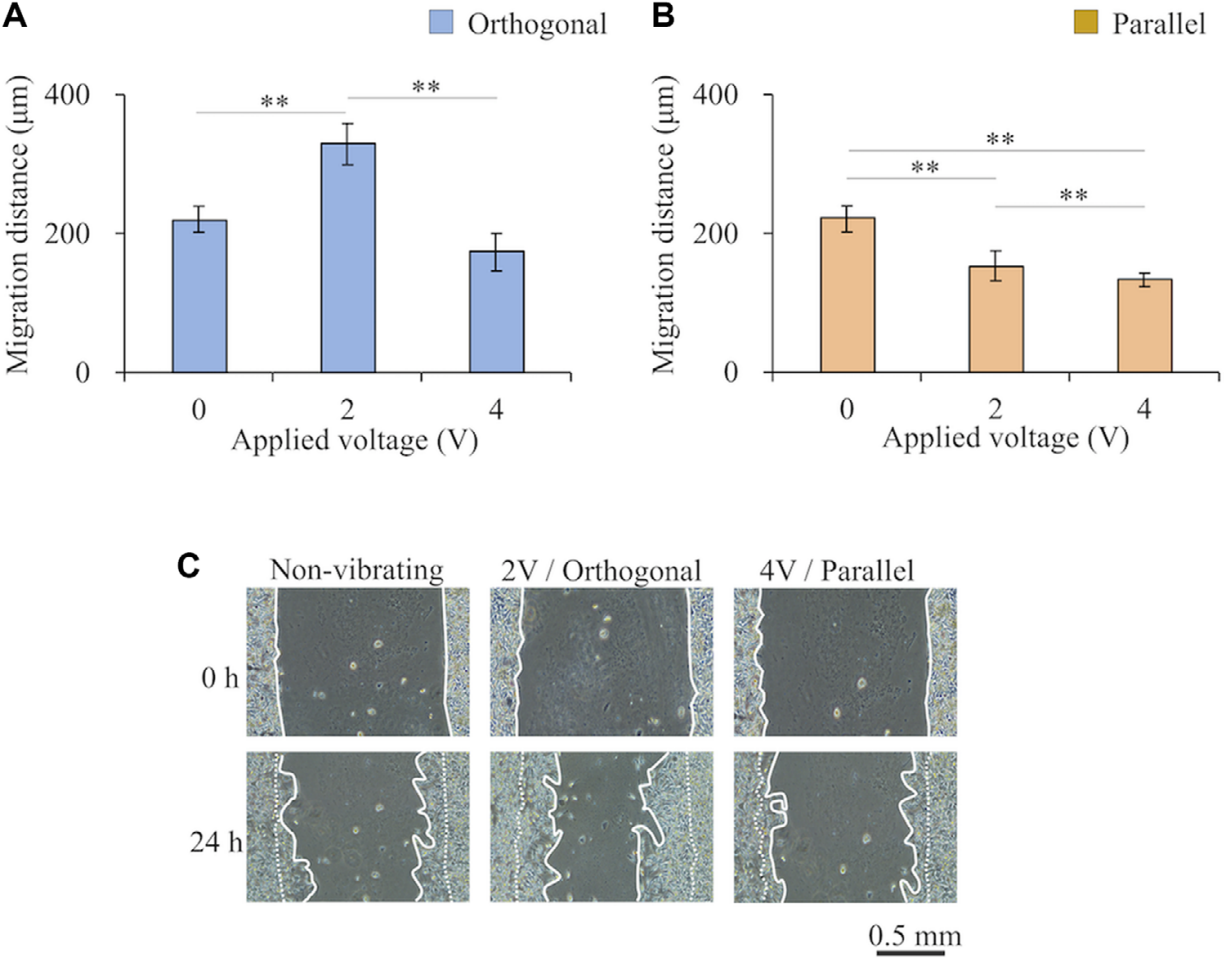
Migration distance results: (A‐B) Migration distances when vibrational stimulation was applied (A) orthogonal to the gap and (B) parallel to the gap. (C) Cell migration over 24 h under three conditions; no vibration (referred to as “non‐vibrating”), vibration orthogonal to the gap with a voltage amplitude of 2 V (“2 V/Orthogonal”), vibration parallel to the gap with a voltage amplitude of 4 V (“4 V/Parallel”). The dashed and solid white lines represent the edges of the cell areas before and after migration. Data are presented as mean ± SD, **p < 0.01, *n* = 3

Cells were subjected to vibrational stimuli in two different directions: orthogonal and parallel to the gap. The frequency of the applied voltage was 11.2 kHz, and the voltage amplitude was 0 (i.e. the no‐vibration condition), 2, and 4 V. The migration distances under all conditions are shown in Figure 4A–C shows the cell migration under three noteworthy conditions that resulted in the greatest absolute differences in migration distance: no vibration (referred to as “non‐vibrating”), vibration orthogonal to the gap with a voltage amplitude of 2 V (“2 V/Orthogonal”), vibration parallel to the gap with a voltage amplitude of 4 V (“4 V/Parallel”). The data shown in Figure 4 indicate that the migration distance varies with the applied voltage in each vibration direction. Moreover, with an applied voltage of 2 V, vibration in the direction orthogonal to the gap resulted in increased migration distance while vibration in the direction parallel to the gap resulted in lower migration distance.

### 3.3 Glucose consumption assay

**FIGURE 5:**
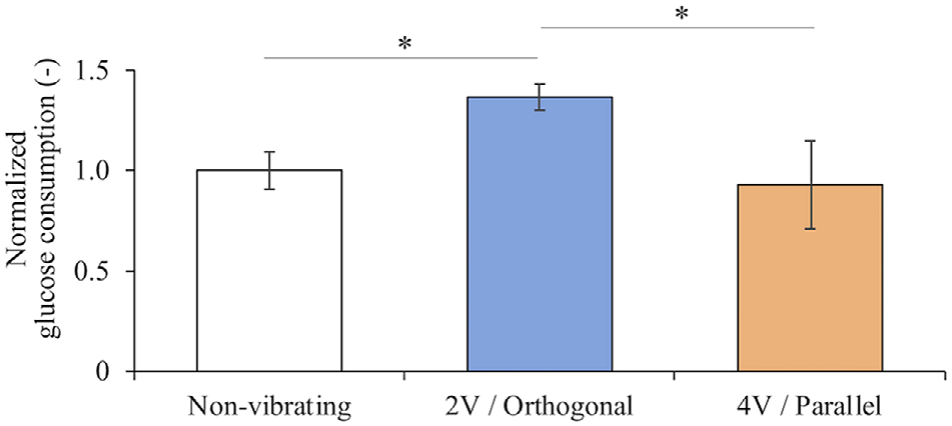
Normalized glucose consumption for 24 h under three conditions; no vibration (referred to as “non‐vibrating”), vibration orthogonal to the gap with a voltage amplitude of 2 V (“2 V/Orthogonal”), vibration parallel to the gap with a voltage amplitude of 4 V (“4 V/Parallel”). Data are presented as mean ± SD, *p < 0.05, *n* = 3

The glucose consumption by cells under three representative and noteworthy vibration conditions that resulted in the greatest absolute differences in migration distance is shown in Figure 5. Glucose consumption was increased under conditions that were associated with the enhanced migration distance relative to the controls that were not exposed to vibration. In contrast, glucose consumption was not changed under conditions that were associated with the most significantly reduced migration distance.

### 3.4 Image‐based quantification

**FIGURE 6:**
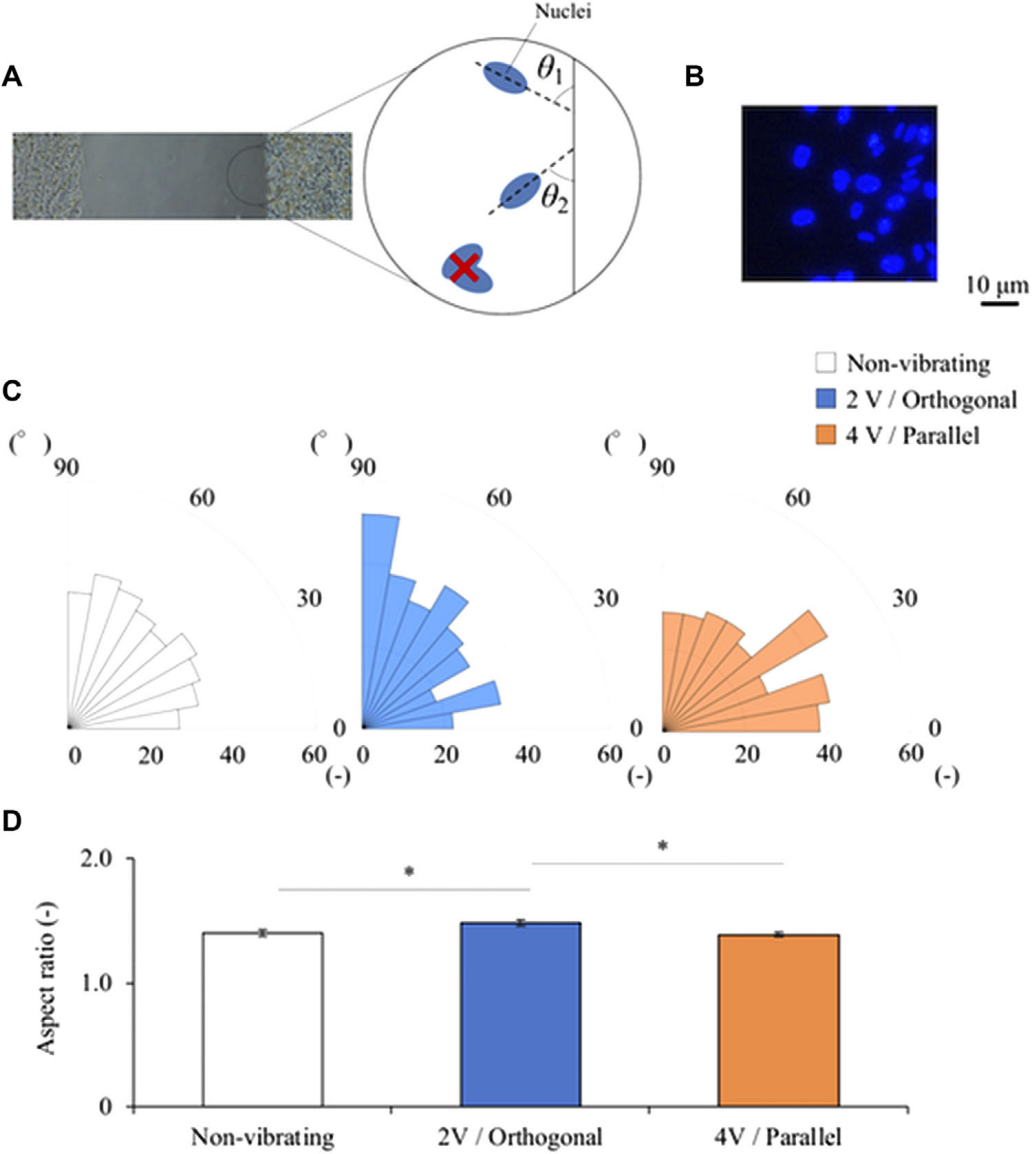
Effect of vibration on cell nuclei orientation: (A) Definition of the angle of a cell nucleus. (B) Nuclear staining image. (C) Orientation angle distributions of the cell nuclei under three conditions. (D) Aspect ratios of cell nuclei; no vibration (referred to as “non‐vibrating”), vibration orthogonal to the gap with a voltage amplitude of 2 V (“2 V/Orthogonal”), vibration parallel to the gap with a voltage amplitude of 4 V (“4 V/Parallel”). *n* = 300

The orientation angle distributions and aspect ratios of the cell nuclei under three noteworthy conditions were measured using Image J. The angle values were expressed in the range of 0 to 90° (acute angles), where orientation angles closer to 90° indicate that cells are oriented in a direction orthogonal to the gap, as illustrated for *θ_1_* and *θ_2_* in Figure 6A. Cases in which multiple cell nuclei overlapped were excluded. The results shown in Figure 6B indicate that the cell nuclei become oriented in the direction orthogonal to the gap under vibration conditions resulting in higher migration distance and, in contrast, become oriented in the direction parallel to the gap under vibration conditions resulting in lower migration distance. The aspect ratios of the cell nuclei under the same conditions are shown in Figure 6C. The results show that orthogonal vibration with an applied voltage amplitude of 2 V caused the nuclei to elongate (as evidenced by the increased aspect ratios) compared with the controls not exposed to vibration. However, parallel vibration with a voltage amplitude of 4 V did not affect the nucleus morphology.

## 4 DISCUSSION

In this study, fibroblasts in a wound model were subjected to horizontal vibration in a custom‐fabricated system to study the effects on collective cell migration. Results showed that the vibration direction affects the cell migration distance, either enhancing or suppressing migration depending on the vibration conditions. Under certain conditions, the cell nuclei became elongated and oriented in the direction of the vibration. In addition, glucose consumption was increased in the conditions under which the migration distance was enhanced. To our knowledge, this is the first study to demonstrate the regulation of collective cell migration by horizontal vibration.

Our results revealed that the direction of the migration regulation depends on the vibrational stimulation conditions. First, we focus on the effects of vibrational stimuli applied orthogonal to the gap (Figure 4A). The migration distance was enhanced with an applied voltage of 2 V compared with the control not exposed to vibration. This effect may be caused by enhanced actin remodeling in response to this stimulus. Actin remodeling is a major factor integral in cell migration and has previously been shown to be upregulated in response to tension applied to the actin filaments [22].

Tension also causes cells to orient in a direction parallel to the applied force, making it easier for the cells to migrate in that direction [13]. Furthermore, the cell nuclei became elongated and oriented in the direction orthogonal to the gap under this condition (Figure 6). These changes can be attributed to the transmission of the applied tensile forces to the nucleus via connected actin filaments, so the change in the aspect ratio and orientation of the cell nuclei may be further evidence of actin remodeling [23].

The observed increased migration in the direction of the vibration is comparable to the effect of other mechanical stimuli. Laminar medium flow has been previously reported to enhance the cell migration distance in the direction of the flow because of the shear forces on the cells [24]. In our experiments, cells were subjected to periodic acceleration, which may induce inertial forces. The direction of these inertial forces likely determines the direction of cell migration, as do the shear stresses induced by fluid flow. As supplementary material, experimental results at different frequencies are described. Figure S1 shows the characteristics of the system, and Figure S2 shows the migration distance under each condition. Similar results were obtained when the magnitudes of the inertial forces were equal, even though the frequencies were different. Note that, the applied voltage was determined so that the magnitudes of the inertial forces were equal to the main experiment. The results show that the key factor affecting migration is the inertial force generated by the vibration. Considering this, the mechanism enhancing migration distance with vibration may be similar to the mechanism with flow stimuli. In short, the cells perceive the force applied to the focal adhesion, which activates Rac, a type of G‐proteins. Then, Rac activates actin polymerization and promotes lamellipodial formation along the force direction. As a result, migration distance in the direction along the force is enhanced.

However, the results showed that the cell migration distance was not increased when the applied voltage was 4 V even though the vibration direction was orthogonal to the gap. This result suggests that vibration with high acceleration has the effect of suppressing migration even when the vibration direction is orthogonal to the gap. It has been previously reported that cells exposed to strong disturbed medium flow form larger focal adhesions to anchor themselves, which inhibits cell migration [14]. Thus, excessive vibrational stimulation due to high voltage input might inhibit migration. Especially when the share stress induced at a focal adhesion due to the vibrational inertia force becomes too large, the vibration suppresses cell adhesion. In other words, vibration whose intensity is beyond a particular threshold suppresses the generation of focal adhesion or breaks focal adhesion [25]. Since cell adhesion to a culture surface is essential in cell migration, it can be reasonable to conclude that migration distance was suppressed by the vibration generated with high voltage input.

Next, we focus on the effects of vibrational stimuli in the direction parallel to the gap. Under these vibration conditions, the migration distance decreased with increasing applied voltage from 0 to 2 V. This relationship with migration distance is opposite that observed with orthogonal vibration. This effect was attributed to the previously mentioned effects of vibration on actin, that is, even though actin remodeling and orientation of cells are affected by parallel vibration, there is no space for cells in the intended direction to migrate. The migration distance was further reduced when the applied voltage was increased from 2 to 4 V, which may be attributed to the formation of focal adhesions in response to excessive vibrational stimulation, as observed with orthogonal vibration.

In addition, the glucose consumption was measured under the same three noteworthy conditions. Results showed that the glucose consumption was elevated under conditions that enhanced the migration distance, as shown in Figure 5. Mechanical stimuli, such as tension and shear stress, are known to increase glucose uptake and ATP production [26] to generate energy as it is consumed to drive actin polymerization and focal adhesion growth, which are necessary for cell migration [27]. Our findings that proper horizontal vibrational stimulation increases glucose consumption are consistent with previous findings with other types of mechanical stimuli. Furthermore, this result indicates that the energy‐consuming activity of the cell was activated. This suggests that cells were neither simply pushed mechanically nor reattached after detachment but were rather promoted migration. The observation that glucose consumption does not decrease under conditions that reduce the migration distance suggests that these conditions do not negatively impact cell viability.

Finally, we focus on the orientation of the cells. The cells subjected to horizontal vibration tended to orient in the direction of vibration under the same three noteworthy conditions. Similar tendency of cell orientation was reported in previous studies using horizontal vibration [19, 28]. However, the results of our studies may have a weaker tendency for cells to orient in the direction of vibration than previous studies. There may be several reasons for this. The first reason is the vibration frequency. A kHz‐order vibration suppresses medium sloshing compared to a Hz‐order vibration. This means, the shear stress applied to cells due to flow in this study may be smaller than in previous studies. In other words, an essential effect of horizontal vibration on cell migration can be extracted in this study. The second reason is a cell density. Due to the high cell density in the wound model, cell deformation and orientation might be inhibited.

These experimental results provide new insights into the regulation of migration by mechanotransduction.

## CONFLICT OF INTEREST

The authors have declared no conflict of interest.

## Supporting information

Fig S1. Characteristics of the fabricated system: (A) Relationship between the applied voltage and the vibration amplitude at *x_1_* = 0 under an applied frequency of 22.4 kHz and amplitude of the applied voltage was 0 to 4.5 V. (B) Vibration amplitude distribution of the dish holder along the *x_1_*, *x_2_*, and *x_3_* axes when the frequency and amplitude of the applied voltage were 22.4 kHz and 2.5 V, respectively.

Fig S2. Migration distance results under an applied frequency of 22.4 kHz: (A‐B) Migration distances when vibrational stimulation was applied (A) orthogonal to the gap and (B) parallel to the gap. Data are presented as mean ± standard deviation, **p < 0.01, *n* = 3.

## References

[1] Friedl, P. , Gilmour., D. , Collective cell migration in morphogenesis, regeneration and cancer. Nat. Rev. Mol. Cell Biol. 2009, 10, 445–57. 19546857 10.1038/nrm2720

[2] Lauffenburger, D. A. , Horwitz., A. F. , Cell migration, A physically integrated molecular process. Cell 1996, 84, 359–369. 8608589 10.1016/s0092-8674(00)81280-5

[3] Weijer, C. J. , Collective cell migration in development. J. Cell Sci. 2009, 122, 3215–3223. 19726631 10.1242/jcs.036517

[4] Lee, S. , Kim, M. S. , Jung, S. J. , Kim, D. , et al., ERK activating peptide, AES16‐2M promotes wound healing through accelerating migration of keratinocytes. Sci. Rep. 2018, 8, 1–10. 30258088 10.1038/s41598-018-32851-y PMC6158248

[5] Masui, T. , Doi, R. , Mori, T. , Toyoda, E. , et al., Metastin and its variant forms suppress migration of pancreatic cancer cells. Biochem. Biophys. Res. Commun. 2004, 315, 85–92. 10.1016/j.bbrc.2004.01.021 15013429

[6] Chung, C. Y. , Funamoto, S. , Firtel, R. A. , Signaling pathways controlling cell polarity and chemotaxis. Trends Biochem. Sci. 2001, 26, 557–66. 11551793 10.1016/s0968-0004(01)01934-x

[7] Bunn, S. J. , Lai, A. , Li, J. , DC electric fields induce perpendicular alignment and enhanced migration in schwann cell cultures. Ann. Biomed. Eng. 2019, 47, 1584–1595. 30963382 10.1007/s10439-019-02259-4

[8] Ross, T. D. , Coon, B. G. , Yun, S. , Baeyens, N. , et al., Integrins in mechanotransduction. Curr. Opin. Cell Biol. 2013, 25, 613–618. 23797029 10.1016/j.ceb.2013.05.006 PMC3757118

[9] Aguilar‐Cuenca, R. , Juanes‐García, A. , Vicente‐Manzanares, M. , Myosin II in mechanotransduction: Master and commander of cell migration, morphogenesis, and cancer. Cell. Mol. Life Sci. 2014, 71, 479–492. 23934154 10.1007/s00018-013-1439-5 PMC11113847

[10] Mitra, S. K. , Hanson, D. A. , Schlaepfer, D. D. , Focal adhesion kinase: In command and control of cell motility. Nat. Rev. Mol. Cell Biol. 2005, 6, 56–68. 15688067 10.1038/nrm1549

[11] Ciccone, E. , Med, J. E. , Mechanotransduction across the cell surface and through the cytoskeleton. Science 2016, 260, 1124–1127. 10.1126/science.7684161 7684161

[12] Alenghat, F. J. , Ingber, D. E. , Mechanotransduction : All signals point to cytoskeleton, matrix, and integrins. Sci. STKE 2002, 119, 1–5. 10.1126/stke.2002.119.pe6 11842240

[13] Lee, J. , Ishihara, A. , Oxford, G. , Johnson, B. , et al., Regulation of cell movement is mediated by stretch‐activated calcium channels. Nature 1999, 400, 382–386. 10432119 10.1038/22578

[14] Hsu, P. P. , Li, S. , Li, Y. S. , Usami, S. , et al., Effects of flow patterns on endothelial cell migration into a zone of mechanical denudation. Biochem. Biophys. Res. Commun. 2001, 285, 751–759. 11453657 10.1006/bbrc.2001.5221

[15] Imashiro, C. , Kurashina, Y. , Kuribara, T. , Hirano, M. , et al., Cell patterning method on a clinically ubiquitous culture dish using acoustic pressure generated from resonance vibration of a disk‐shaped ultrasonic transducer. IEEE Trans. Biomed. Eng. 2019, 66, 111–118. 29993416 10.1109/TBME.2018.2835834

[16] Jang, K. W. , Ding, L. , Seol, D. , Lim, T. ‐. H. , et al., Low‐intensity pulsed ultrasound promotes chondrogenic progenitor cell migration via focal adhesion kinase pathway. Ultrasound Med. Biol. 2014, 40, 1177–86. 24612644 10.1016/j.ultrasmedbio.2013.12.007 PMC4034572

[17] Mohammed, T. , Murphy, M. F. , Lilley, F. , Burton, D. R. , et al., The effects of acoustic vibration on fibroblast cell migration. Mater. Sci. Eng. C 2016, 69, 1256–1262. 10.1016/j.msec.2016.07.037 27612824

[18] Ao, M. , Brewer, B. M. , Yang, L. , Franco Coronel, O. E. , et al., Stretching fibroblasts remodels fibronectin and alters cancer cell migration. Sci. Rep. 2015, 5, 8334. 25660754 10.1038/srep08334 PMC4321168

[19] Halonen, H. T. , Ihalainen, T. O. , Hyväri, L. , Miettinen, S. , et al., Cell adhesion and culture medium dependent changes in the high frequency mechanical vibration induced proliferation, osteogenesis, and intracellular organization of human adipose stem cells. J Mech Behav Biomed Mater 2020, 101, 103419. 31518945 10.1016/j.jmbbm.2019.103419

[20] Riahi, R. , Yang, Y. , Zhang, D. D. , Wong., P. K. , Advances in wound‐healing assays for probing collective cell migration. J. Lab. Autom. 2012, 17, 59–65. 22357609 10.1177/2211068211426550

[21] Wiklund, M. , Acoustofluidics 12: Biocompatibility and cell viability in microfluidic acoustic resonators. Lab Chip 2012, 12, 2018–2028. 22562376 10.1039/c2lc40201g

[22] Gardel, M. L. , Schneider, I. C. , Aratyn‐Schaus, Y. , Waterman, C. M. , Mechanical integration of actin and adhesion dynamics in cell migration. Annu. Rev. Cell Dev. Biol. 2010, 26, 315–333. 19575647 10.1146/annurev.cellbio.011209.122036 PMC4437624

[23] Maniotis, A. J. , Chen, C. S. , Ingber, D. E. , Demonstration of mechanical connections between integrins, cytoskeletal filaments, and nucleoplasm that stabilize. Proc. Natl. Acad. Sci. 2000, 94, 849–854. 10.1073/pnas.94.3.849 PMC19602 9023345

[24] Li, S. , Huang, N. F. , Hsu., S. , Mechanotransduction in endothelial cell migration. J. Cell. Biochem. 2005, 96, 1110–1126. 16167340 10.1002/jcb.20614

[25] Inui, T. , Kurashina, Y. , Imashiro, C. , Takemura, K. , Method of localized removal of cells using a bolt‐clamped Langevin transducer with an ultrasonic horn. Eng. Life Sci. 2019, 19, 575–583. 32625033 10.1002/elsc.201800173 PMC6999498

[26] Bays, J. L. , Campbell, H. K. , Heidema, C. , Sebbagh, M. , et al., Linking E‐cadherin mechanotransduction to cell metabolism through force‐mediated activation of AMPK. Nat. Cell Biol. 2017, 19, 724–731. 28553939 10.1038/ncb3537 PMC5494977

[27] Cipolla, M. J. , Gokina, N. I. , Osol., G. , Pressure‐induced actin polymerization in vascular smooth muscle as a mechanism underlying myogenic behavior. FASEB J. 2002, 16, 72–76. 11772938 10.1096/cj.01-0104hyp

[28] Pongkitwitoon, S. , Uzer, G. , Rubin, J. , Judex, S. , Cytoskeletal configuration modulates mechanically induced changes in mesenchymal stem cell osteogenesis, morphology, and stiffness. Sci. Rep. 2016, 6, 1–12. 27708389 10.1038/srep34791 PMC5052530

