# Supplementary figures and images for: Collective cell migration of fibroblasts is affected by horizontal vibration of the cell culture dish
Source: Eng Life Sci. 2020 Jul 19;20(9-10):402–11. doi: 10.1002/elsc.202000013 (PMC7481772; doi:10.1002/elsc.202000013)

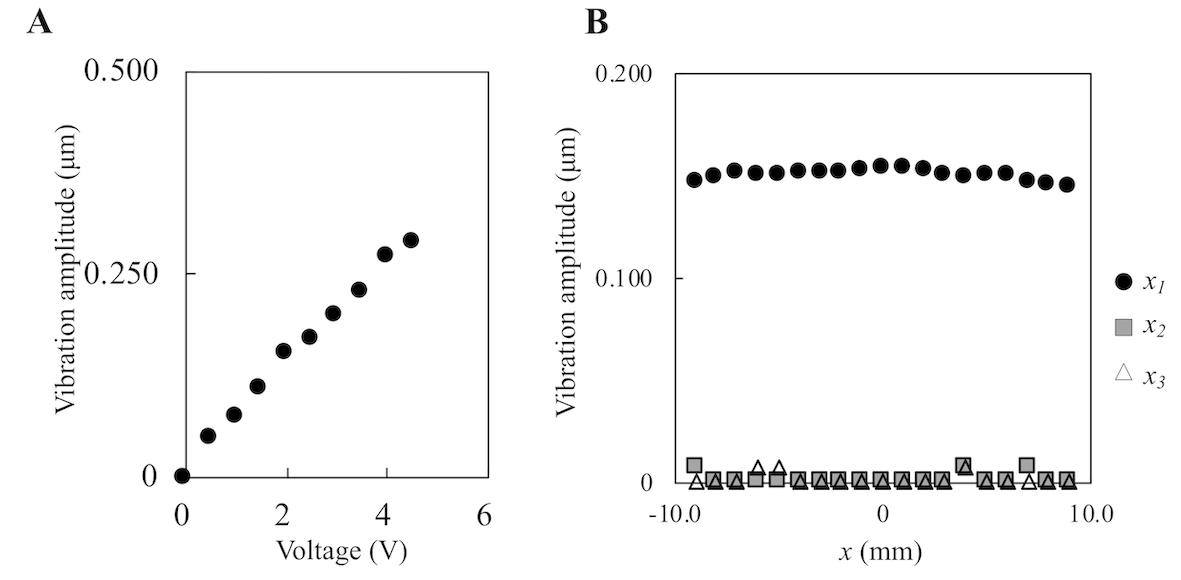

Supplement: Supplementary file 1 — Fig S1. Characteristics of the fabricated system: (A) Relationship between the applied voltage and the vibration amplitude at x1 = 0 under an applied frequency of 22.4 kHz and amplitude of the applied voltage was 0 to 4.5 V. (B) Vibration amplitude distribution of the dish holder along the x1, x2, and x3 axes when the frequency and amplitude of the applied voltage were 22.4 kHz and 2.5 V, respectively. [file ELSC-20-402-s002.tiff]

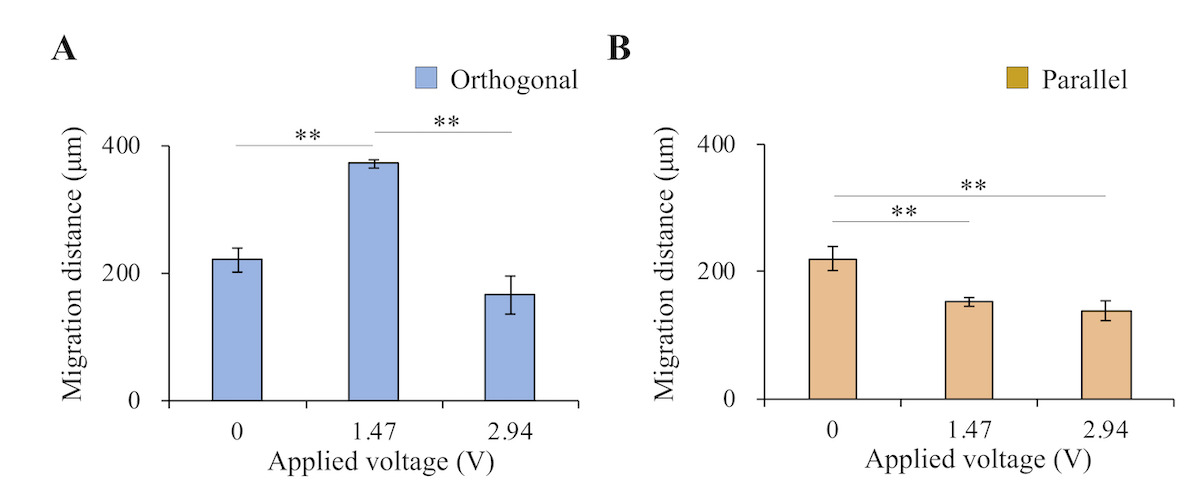

Supplement: Supplementary file 2 — Fig S2. Migration distance results under an applied frequency of 22.4 kHz: (A‐B) Migration distances when vibrational stimulation was applied (A) orthogonal to the gap and (B) parallel to the gap. Data are presented as mean ± standard deviation, **p < 0.01, n = 3. [file ELSC-20-402-s001.tiff]
